# Characterizing tobacco and marijuana use among youth combustible tobacco users experiencing homelessness – considering product type, brand, flavor, frequency, and higher-risk use patterns and predictors

**DOI:** 10.1186/s12889-022-13244-3

**Published:** 2022-04-25

**Authors:** Allison M. Glasser, Alice Hinton, Amy Wermert, Joseph Macisco, Julianna M. Nemeth

**Affiliations:** 1 Division of Health Behavior and Health Promotion, The Ohio State University College of Public Health, 1841 Neil Avenue, Columbus, OH 43210 USA; 2 Division of Biostatistics, The Ohio State University College of Public Health, 1841 Neil Avenue, Columbus, OH 43210 USA

**Keywords:** Homelessness, Youth, Young adults, Combustible, Marijuana, Poly-tobacco

## Abstract

**Background:**

Cigarette smoking is three times more prevalent among youth experiencing homelessness compared with the general population. Co-use of tobacco and marijuana is also common. The aim of this study is to characterize tobacco and marijuana use among youth experiencing homelessness who use combustible tobacco in a Midwestern city to inform smoking cessation intervention.

**Methods:**

This study included 96 youth (ages 14–24 years; 52% male, 39% female, 5% transgender/non-binary) attending a homeless drop-in center who had used at least one combustible tobacco product in the past week. We assessed past-month use of tobacco products and marijuana, other product use characteristics (e.g., frequency, brand and flavor), and psychosocial predictors of more frequent (i.e., daily) use of combustible tobacco and marijuana.

**Results:**

Most youth experiencing homelessness with past-week combustible tobacco use had used cigarettes (*n* = 85, 88.5%), cigars (*n* = 89, 92.7%), and marijuana (*n* = 82, 85.4%) in the past month. One-third (*n* = 34) used electronic vapor products (EVPs), 19.8% (*n* = 19) smoked hookah, and 11.5% (*n* = 11) used smokeless tobacco (ST). Most marijuana users co-administered with tobacco (*n* = 67, 69.8%). Daily combustible tobacco smoking was associated with having a child and smoking out of boredom/habit. Daily marijuana use was associated with using substances to cope with one’s housing situation. Newport (*n* = 66, 72.5%) and Black & Mild (*n* = 48, 51.1%) were the most popular brands of cigarettes and cigars among ever users. Most non-combustible tobacco ever users reported not having a usual brand (EVPs: *n* = 51, 73.9%; ST: *n* = 16, 57.1%). Cigar smokers reported the most varied selection of flavors.

**Conclusions:**

Young combustible tobacco users experiencing homelessness engage in high-risk use patterns, including poly-tobacco use, co-use of tobacco with marijuana, and frequent combustible product use. Interventions that consider the full context of tobacco and marijuana use are needed to support smoking cessation in this population.

**Supplementary Information:**

The online version contains supplementary material available at 10.1186/s12889-022-13244-3.

## Background

As combustible tobacco smoking has declined nationally in the United States (US) following decades of tobacco control policies and treatments, vulnerable populations have been left behind [1]. Members of these populations continue to smoke at alarming rates and experience resulting health disparities. It is therefore critical to determine how to help these marginalized groups engage in smoking cessation, including increasing motivation to quit, quit attempts using evidence-based methods, and sustained abstinence [2, 3]. For youth experiencing homelessness (YEH), one of the US’s most vulnerable populations, awareness of characteristics of tobacco use and the psychosocial context of smoking behavior can identify opportunities to target intervention strategies and thereby enhance population-specific cessation effectiveness [4, 5].

Cigarette smoking is about three times more prevalent among YEH (ages 14 to 25 years) compared with the general population of youth and young adults [6–8]. Overall, combustible tobacco use, including cigarette and cigar smoking, constitutes the primary tobacco products used in this population [9]. Additionally, electronic vapor products (EVPs) are used among young adults experiencing homelessness at nearly twice the rate and smokeless tobacco about five times the rate of use in the general population of young adults [10–12]. Although YEH are primarily traditional combustible tobacco users or co-use cigarettes and cigars, poly-tobacco use is also prevalent, particularly among those with substance use disorder and among those who have spent more nights outdoors [9].

Substance use overall is extremely high among YEH [13], with estimates up to 96% [14]. In particular, co-administration of tobacco and marijuana (combining the two products, such as in a blunt, where one removes tobacco contents from a cigar and replaces or mixes it with marijuana) is common among YEH. A study of youth and young adults experiencing homelessness in Los Angeles (LA) County, California found that about 90% of tobacco users consumed tobacco and marijuana together [15]. These users were heavier users of tobacco and marijuana compared to those who used tobacco alone or co-used (used both simultaneously or within the same time-period), but not co-administered; they also tended to experience more severe homelessness and other risk factors like depression. Little cigars and cigarillos are commonly viewed among young adults experiencing homelessness as a discreet way to smoke marijuana [16]. About three-quarters of cigar smoking high school youth in Cuyahoga County, Ohio in 2013 reported concurrent past 30-day marijuana use, about half reported “freaking” their cigar (removing the filter paper from the cigar and repacking), and two-thirds reported using blunts [17]. In addition to elucidating patterns of tobacco use among YEH, co-use of marijuana with tobacco needs to be understood as it relates to smoking cessation and its potential need to be addressed when supporting quitting among YEH.

Marijuana and tobacco are often used to cope with stress and traumatic events, which are common among YEH [18, 19]. A study of smokers experiencing homelessness found that a large proportion had experienced trauma and reported posttraumatic stress symptoms; these individuals endorsed smoking to reduce negative affect and for the positive social effects [20]. Tobacco use is socially acceptable and rather ubiquitous in homeless drop-in facilities and shelters, facilitating continued smoking among those in attendance [19]. These factors unique to the homeless experience create a physical and social environment where tobacco use is normal and even expected, creating barriers to smoking cessation [21]. Despite the high prevalence of tobacco use in this population, many YEH are willing to quit smoking. One-fifth to one-third of tobacco product users among youth and young adults experiencing homelessness in LA County were willing to quit their product in 2018 [22]. A previous analysis of qualitative data from the current study showed that while willingness to quit is high, many YEH are not successful at quitting and have limited access to support [23]. It is critical that we develop strategies to get YEH access to evidence-based cessation support and, for those not yet motivated to quit smoking, intervene to move them into a pre-cessation phase where they may begin to utilize cessation support [3]. Research on smoking cessation interventions for this target population is nascent, although some work has shown that providers at shelters and drop-in centers are willing to provide cessation services [24], and engaging smokers outside of a service setting (e.g., via mobile phone) may also be feasible for intervention [25, 26].

Much remains to be understood about tobacco and marijuana use among YEH. A more detailed characterization of tobacco use among homeless youth is needed, including understanding frequency of use, use of flavored tobacco, and what brands these youth are using. Researchers have emphasized the importance of conducting research with YEH that addresses both the basic science of smoking and the factors that influence and maintain smoking behavior [27]. Along these lines, it is critical to better understand the psychosocial context relevant to smokers [28], particularly frequent users of combustible tobacco and marijuana [29], to account for relevant factors when developing combustible tobacco cessation intervention for the highest risk smokers. It is also important to determine how patterns of use compare across samples of YEH assessed in varying geographic settings where prevalence of tobacco use and regulatory contexts differ. For example, most studies based in the US on tobacco use among YEH were conducted in larger cities primarily on the west coast [9, 15, 22].

The overall aim of this study is to quantitatively explore findings from a previous qualitative study that sought to establish a theoretical framework for cessation among YEH, incorporating factors impacting motivation to engage in cessation [23, 30]. Specifically, we aim to characterize tobacco use among YEH who use combustible tobacco in a Midwestern city, including frequency of product use, brand and flavor preferences, co-use with marijuana, and predictors of frequent combustible tobacco and marijuana use. This information will help us to develop targeted smoking cessation interventions, particularly in a drop-in center setting.

## Methods

### Participants

Participants were YEH [31] (ages 14–24 years) in a Midwestern city attending a drop-in center that was established to facilitate health intervention research and provides a safe place for youth to rest, eat, wash clothes, shower, and receive case management and requested treatment services. A total of 139 participants were recruited for this study by being approached for eligibility at the drop-in center by research staff. Participants were eligible if they had used at least one combustible tobacco product in the past week, were not currently making an attempt to quit smoking, were attending a drop-in center, and had not participated in an earlier phase of this study. Thirty-one participants were ineligible, ten participants refused, and two participants partially completed the survey and were excluded due to unreliable responses. The final sample consisted of 96 YEH who consented/assented to participate (a waiver of parental consent was obtained to enroll youth 14–17 years of age) and completed the survey.

### Procedures

The study was conducted in accordance with the Declaration of Helsinki, and the protocol was approved by the Ethics Committee of Ohio State University (#2017C0148). Data were collected through an approximately 90-min interviewer-administered survey from December 2019 through March 2020. Trained research staff read questions to the participants while showing response option cards when appropriate. Responses were recorded directly into Qualtrics by the interviewer; however, more sensitive questions were administered via audio-CASI (computer-assisted self-interviewing), for which the participants entered their own responses. Each participant received a $25 grocery gift card incentive.

### Measures

#### Demographic characteristics

We measured participants’ age, gender (male, female, genderqueer, intersex, transgender female, transgender male, transgender, other), sexual orientation (heterosexual/straight, gay, lesbian, bisexual, queer/questioning, asexual, other), race (American Indian or Alaska Native, Asian, Black or African American, Native American, Native Hawaiian or another Pacific Islander, White, bi- or multi-racial, other), Hispanic ethnicity, education (less than high school, high school diploma, general educational development (GED), more than high school), number of children, pregnancy status for youth assigned female at birth, hours worked per week, and location slept most nights.

#### Ever and past 30-day tobacco product and marijuana use

We measured ever use and past 30-day frequency of use (0 days, 1–2 days, 3–5 days, 6–9 days, 10–19 days, 20–29 days, all 30 days) of the following products: cigarettes, cigars, hookah, EVPs, smokeless tobacco, and marijuana.

#### Usual brand and flavor

We asked participants if they have a usual brand for each product (excluding marijuana) (yes/no; asked of participants who had ever used that product), what that brand is, and whether that brand is usually flavored. Flavor categories were menthol or mint, clove or spice, fruit, chocolate, an alcoholic drink (such as wine, cognac, margarita, piña colada, peach schnapps, or other cocktails), candy or sweets, tobacco, coffee, vanilla, cola, or other [32].

#### Marijuana administration and lifetime use frequency

Among past 30-day marijuana users (at least once in the past 30 days), we measured the usual method of administration (blunt [cigar hallowed out and filled with marijuana]; joint, bong, pipe; spliff [combination of tobacco and marijuana]; food; drink; vaporized; some other way). We also asked participants how many times they have used marijuana in their lifetime (0, 1–2, 3–9, 10–19, 20–39, 40–99, and 100+ times).

#### Other tobacco and psychosocial variables

We assessed a number of tobacco-related and psychosocial factors that may be related to tobacco use and to homeless experiences; these measures were selected based on behavior change theory [33] and on findings from qualitative interviews conducted in an earlier phase of this study [23, 30]. The details of these measures are provided in Supplemental Table 1. Briefly, we assessed tobacco dependence using the Hooked on Nicotine Checklist (HONC; 10-item instrument to identify signals of loss of autonomy among adolescents) [34], first use of tobacco (product, age), and motivations, temptations, and rewards from smoking [35, 36], and alcohol use. In addition, other scales assessed interoceptive awareness (e.g., attention and emotion regulation), strategies to cope with one’s housing situation, and anger/worry management [37–40].

### Data analyses

Categorical variables are summarized with frequencies and percentages while continuous and ordinal variables are summarized with means and standard deviations or medians and interquartile ranges (IQR), as is appropriate based on the distribution of the variable. Fisher exact tests, t tests, and Wilcoxon rank-sum tests were conducted to examine group differences between: 1) daily combustible tobacco users vs. non-daily combustible users, and 2) daily marijuana users vs. non-daily marijuana users. Multivariable logistic regression models were fit to determine independent predictors (demographic, tobacco use, and psychosocial measures described above) of daily combustible use and separately, among the subset of current marijuana users, daily marijuana use. Due to the exploratory nature of these analyses, stepwise selection was used to determine the terms included in the final models. All analyses were conducted in SAS 9.4 (SAS Institute Inc., Cary, NC), and *p*-values < 0.05 were significant.

## Results

### Sample characteristics

Participants were mostly aged 18–24 years (*n* = 93, 97%), identified as male (*n* = 53, 55%), heterosexual (*n* = 71, 74%), Black (*n* = 51, 53%) or multi-racial (*n* = 27, 28%), non-Hispanic (*n* = 88, 92%), and had earned a high school diploma (*n* = 46, 48%) or less than high school (*n* = 31, 32%) (Table 1). Slightly more than one-third had children, and about half were unemployed. Participants were relatively evenly split between sleeping at a friend’s house or own home (*n* = 31, 32%), at the shelter or drop-in center (*n* = 27, 28%), or outside on most nights (*n* = 25, 26%). Participants were, on average, moderately dependent on nicotine (median = 6, IQR = 3.5–8).

**Table 1:** Demographic and Tobacco Use Characteristics of Youth Experiencing Homelessness

	***N*** = 96	
	*n*	*%*
**Age**		
14–17	3	3%
18–24	93	97%
**Age** (mean, SD)	21.82	2.00
**Gender**^**a**^		
Male	52	54%
Female	39	41%
Transgender Female	2	2%
Transgender Male	2	2%
Non-binary	1	1%
**Sexual Orientation**^**b**^		
Heterosexual/Straight	71	74%
Bisexual	19	20%
Other	6	6%
**Race**		
White	15	16%
Black	51	53%
Bi or Multi-racial	27	28%
Other	3	3%
**Ethnicity**		
Non-Hispanic	88	92%
Hispanic	8	8%
**Education**		
Less than High School	31	32%
High School Diploma	46	48%
GED	4	4%
More than High School	15	16%
**Children**		
None	55	57%
1 or more	41	43%
**Currently Pregnant**		
No	36	86%
Yes	3	7%
Don’t Know	3	7%
**Hours Work per Week**		
0	50	52%
1–39	20	21%
≥ 40	24	25%
**Where Slept Most Nights**		
With family or friends / Own home	31	32%
Shelter / Drop-in-center	27	28%
Group home / Treatment facility / Detention facility	13	14%
Outside / Car / Tent	25	26%
**Tobacco Use**		
Single Combustible	10	11%
Poly Combustible, no EVP/ST	48	51%
Combustible / EVP	26	27%
Combustible / ST	3	3%
Combustible / EVP/ ST	8	8%
**Tobacco / Marijuana Use**		
Tobacco Only	14	15%
Comb. / Marijuana	48	50%
Comb. / EVP / Marijuana	24	25%
Comb. / ST / Marijuana	3	3%
Comb. / EVP / ST / Marijuana	7	7%
**HONC** (median, IQR)	6	(3.5–8)

*GED* General Education Development, *SD* standard deviation, *EVP* electronic vapor product, *IQR* interquartile range, *HONC* Hooked on Nicotine Checklist

^a^ No participants identified as intersex or genderqueer, so they are not included

^b^ No participants identified as gay, lesbian, queer/questioning, or asexual, so they are not included

### Ever and past 30-day (current) product use

#### Combustible tobacco

**Fig. 1:**
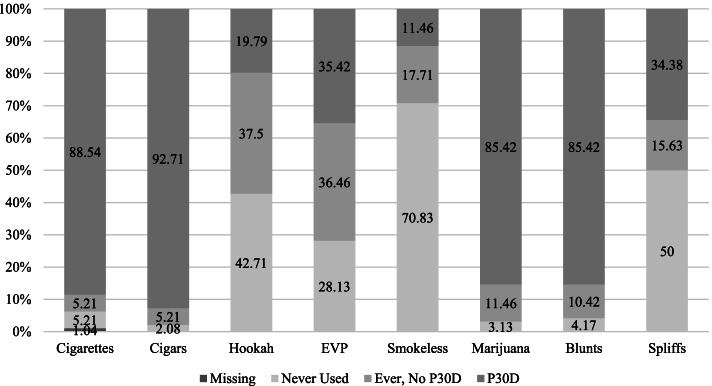
Never, Ever, and Past 30-Day Use of Tobacco Products and Marijuana^a^. ^a^ A blunt is a cigar hallowed out and filled with marijuana; a spliff is a combination of tobacco and marijuana. EVP: electronic vapor product; P30D: past 30-day use

Among the full sample, the most common product used was cigars, with 92.7% (*n* = 89) smoking cigars currently, only 5.2% (*n* = 5) having tried them but not currently using them, and 2.1% (*n* = 2) having never smoked them (Fig. 1). Closely following cigars, 88.5% (*n* = 85) smoked cigarettes currently, and equal proportions of the sample had ever smoked cigarettes (but did not currently smoke cigarettes) and had never smoked cigarettes (*n* = 5, 5.2%). Most of the youth in our sample did not report currently smoking hookah (ever tried, but not currently smoking: 37.5% (*n* = 36), never tried hookah: 42.7% (*n* = 41)), although about one-fifth of the sample did report currently smoking hookah (*n* = 19, 19.8%).

#### Non-combustible tobacco

Seventy-two percent (*n* = 69) of youth in our sample ever used an EVP, while less than one-third (*n* = 28, 29.2%) had ever used a smokeless tobacco product. Current use was lower where just over one-third (*n* = 34) of the youth in this study used EVPs and 11.5% (*n* = 11) used smokeless tobacco in the past month (Fig. 1).

#### Marijuana

Most youth in the sample were currently using marijuana (85.4% (*n* = 82) marijuana overall; 85.4% blunts (*n* = 82); Fig. 1). Thirty-four percent (*n* = 33) of the sample were currently using spliffs. Almost three-quarters (*n* = 69, 71.9%) of the youth in this study reported having used marijuana more than 100 times in their lives, followed by 12.5% (*n* = 12) reporting 40–99 times and 5.2% (*n* = 5) 20–39 times. Most marijuana users usually co-administered with tobacco, with 67.7% (*n* = 65) of our sample usually smoking blunts and 2.1% (*n* = 2) usually smoking spliffs. An additional 11.5% (*n* = 11) reported usually smoking marijuana in a joint, bong or pipe, and 2.1% (*n* = 2) reported usually smoking marijuana in a bowl.

#### Poly-product use

The primary use patterns (Table 1) in the past month among YEH in this sample was poly-combustible use (*n* = 48, 51%) or combustible plus EVP use (*n* = 26, 27%). Only 8% (*n* = 8) used all tobacco product types we assessed, and about 11% (*n* = 10) used just one combustible product. Few (*n* = 3, 3%) used smokeless tobacco without EVPs.

Similar to strictly tobacco use patterns, about half of the sample reported concurrent use of combustible tobacco and marijuana (*n* = 48), and about one quarter (*n* = 24) reported concurrent use of combustible tobacco, marijuana, and EVPs (Table 1). Fifteen percent (*n* = 14) used only tobacco with no marijuana, and 7% (*n* = 7) used all product types assessed.

### Frequency of product use in the past 30 days

**Fig. 2:**
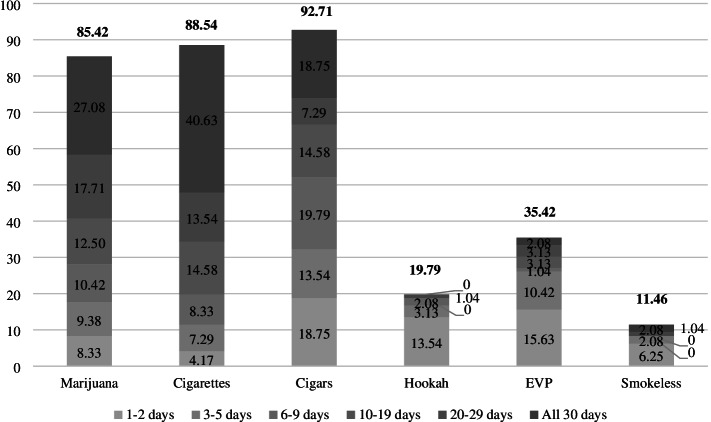
Past 30-Day Marijuana and Tobacco Product Use Frequency. EVP: electronic vapor product

Combustible products (except hookah) were more frequently used than non-combustible products in this study. The most frequently used product in the past month among youth in our sample was cigarettes, with 40.6% (*n* = 39) reporting daily use (Fig. 2), followed by marijuana (*n* = 26, 27.1%), cigars (*n* = 18, 18.8%), and EVPs and smokeless tobacco (*n* = 2, 2.1% each). No one reported daily hookah smoking. Infrequent use (1–2 days) was most common among cigar smokers (*n* = 18, 18.8%), followed by EVPs (*n* = 15, 15.6%), hookah (*n* = 13, 13.5%), marijuana (*n* = 8, 8.3%), smokeless tobacco (*n* = 6, 6.3%), and cigarettes (*n* = 4, 4.2%).

#### Daily vs. non-daily combustible use

We examined differences between daily and non-daily combustible tobacco users (Supplemental Table 2). Compared to non-daily combustible tobacco use, daily combustible tobacco use was associated with having at least one child (55.6% vs. 31.4%, *p* = 0.017), higher mean nicotine dependence as measured by the HONC (6.5 vs. 5.2, *p* = 0.039), past 30-day cigarette smoking (97.8% vs. 82.0%, *p* = 0.039), poly-combustible use (60.0% vs. 42.0%, *p* = 0.006), and younger mean age when first tried tobacco (13 years vs. 15 years, *p* = 0.028). In addition, daily combustible tobacco users were more likely than non-daily users to endorse smoking for boredom relief (*p* < 0.001), stress relief (*p* = 0.025), or because it’s comforting (*p* = 0.002), and smoking related to negative affect (*p* = 0.024) or habit (*p* = 0.001). More daily combustible tobacco users also reported binge drinking in the past month (*p* = 0.043).

In multivariable analyses (Table 2), significant predictors of daily combustible tobacco use were having at least one child (odds ratio (OR) = 3.52, 95% confidence interval (CI) = 1.25, 9.92), smoking related to habit (OR = 4.00, 95% CI = 1.08, 14.83), smoking for boredom relief (OR = 1.40, 95% CI = 1.14, 1.71), and higher scores on the non-distracting scale of the Science of Behavior Change (SOBC) Multidimensional Assessment of Interoceptive Awareness (MAIA) measure (OR = 1.67, 95% CI = 1.10, 2.54), indicating that those who tend not to ignore or distract oneself from sensations of pain or discomfort had higher odds of being a daily smoker.

**Table 2:** Multivariable logistic regression models assessing relationship between tobacco/psychosocial factors and daily combustible/marijuana use

	Daily (vs. Non-Daily) Combustible (***n*** = 45; 47%)			Daily (vs. Non-Daily) Marijuana (***n*** = 26; 30%)		
Psycho-Social Factors	OR	95% CI	***p*** value	OR	95% CI	***p*** value
≥1 child (vs. no children)	3.52	(1.25–9.92)	0.017	–	–	–
Situational Temptations Inventory: Habit (*1-unit increase*)	4.00	(1.08–14.83)	0.038	–	–	–
Motivations for Smoking: Boredom Relieve (*1-unit increase*)	1.40	(1.14–1.71)	0.001	–	–	–
SOBC MAIA: Non-Distracting (*1-unit increase*)	1.67	(1.10–2.54)	0.016	–	–	–
Situational Temptations Inventory: Negative Affect (*1-unit increase*)	–	–	–	0.03	(0.004–0.21)	0.001
SOBC Brief Cope: Substance (*1-unit increase*)	–	–	–	1.72	(1.19–2.48)	0.004
Age First Tried Tobacco (*5-unit increase*)	–	–	–	0.25	(0.10–0.64)	0.004

*OR* odds ratio, *CI* confidence interval, *SOBC* Science of Behavior Change, *MAIA* Multidimensional Assessment of Interoceptive Awareness

#### Daily vs. non-daily marijuana use

There were also differences between daily and non-daily marijuana users (*n* = 82). In univariable analyses (Supplemental Table 3), compared to non-daily marijuana users, daily marijuana users had lower mean nicotine dependence (4.7 vs. 6.4, *p* = 0.017) and were less likely to report that a combustible tobacco product was their first tobacco product tried (84.6% vs. 98.2%, *p* = 0.048). Daily marijuana use was associated with reporting smoking (combustible tobacco) related to the positive social aspects (*p* = 0.045), but less so when experiencing negative affect (*p* = 0.015) and because it is calming (*p* = 0.045). Daily marijuana use was associated with lower scores on the dysregulation scale of the SOBC Children’s Emotion Management Scale (CEMS): Worry measure, indicating that daily marijuana users may self-regulate worry better than non-daily users (*p* = 0.045).

In multivariable analyses (Table 2), those who reported that they smoke combustible tobacco related to experiencing negative affect had lower odds (OR = 0.03, 95% CI = 0.004, 0.21) of daily marijuana use. The odds of being a daily marijuana user decreased by 75% for every five-year increase in age of first trial of tobacco (*p* = 0.004). Those who reported using substances to feel better about or get through one’s housing situation had 72% higher odds of being a daily marijuana user (*p* = 0.004).

### Usual brand and flavor

Most (*n* = 81, 89.0%) cigarette smokers reported a usual brand (Supplemental Fig. 1). Almost three-quarters (*n* = 66, 72.5%) of cigarette smokers reported that their usual brand was Newport, followed by Marlboro (*n* = 5, 5.5%). The remainder of the brands reported made up 2% or less of cigarette smokers. Eighty-one percent (*n* = 76) of cigar smokers reported a usual brand. About half (*n* = 48, 51.1%) of cigar smokers reported that their usual brand was Black & Mild, followed by Swisher Sweets (*n* = 15, 16.0%). The remainder of the brands reported made up 2% or less of cigar smokers. Most EVP users did not report a usual brand (*n* = 49, 72%), 8.7% (*n* = 6) reported usually using JUUL, and 7.2% (*n* = 5) did not know what brand they usually used. Almost 60% (*n* = 12) of smokeless tobacco users did not report a usual brand. Twenty-five percent (*n* = 7) of smokeless tobacco users reported usually using Grizzly, 10.7% (*n* = 3) Copenhagen, and 7.1% (*n* = 2) other brands.

Among cigarette smokers who reported a usual brand (*n* = 81), 72.8% (*n* = 59) usually smoked a menthol or mint flavored brand (Supplemental Table 4). Cigar smokers reported the most varied selection of flavored products. About half (*n* = 37) of cigar smokers who report a usual brand (*n* = 76) use flavored products, ranging from 1.3% (*n* = 1) vanilla and coffee to 15.8% (*n* = 12) fruit. Almost all EVP users who reported a usual brand (*n* = 18) reported using flavored products; over half (*n* = 10) usually used fruit flavored EVPs. Most (*n* = 9, 75%) of the smokeless tobacco users who had a usual brand (*n* = 12) used a menthol or mint flavor, while 25.0% (*n* = 3) used fruit flavors.

## Discussion

The aim of this study was to characterize tobacco use, including co-use with marijuana, poly-tobacco, flavor, and brand use, frequency of product use, and predictors of frequent use, among YEH who use combustible tobacco in a Midwest city to inform combustible tobacco cessation intervention.

Findings indicate that most (85%) combustible tobacco users in our study currently used marijuana, used marijuana on ≥100 occasions in their lives (72%), and co-administered marijuana with tobacco (e.g., blunt, spliff; 70%). These findings are consistent with another larger study of YEH in LA County that found 90% of any tobacco users were also using marijuana and 65% were co-administering tobacco and marijuana [15]. Nationally in the US, among high school-aged youth, 53.6% of single tobacco product (cigarettes, cigars, smokeless tobacco) users and 64.5% of users of at least two tobacco products also used marijuana in the past month [41]. Together, these studies suggest that co-use of marijuana may be more common among YEH than the general population of young people.

In addition to concurrent use of tobacco and cannabis, poly-tobacco use was common in our study, with 89% reporting the use of a combustible product and at least one other product. Poly-tobacco use in the general population of youth and young adults is lower than what was observed in our study. In 2013, 57.1% of youth and 65.2% of young adults who used cigarettes also used at least one other product in the past month [42], and over 70% of past-month cigar smokers used at least one other tobacco product [43]. The most prevalent combination of products used in our study was two or more combustible products. Combustible products (except hookah) were more frequently used than non-combustible products. Perhaps relatedly, brand preferences were more common for cigarettes and cigars than for smokeless tobacco and EVPs. Newport and Black & Mild were the most popular cigarette and cigar brands, respectively. Consistent with the popularity of Newport cigarettes, most (three-quarters) cigarette smokers usually smoked menthol cigarettes. About half of cigar smokers usually use a flavored product, with fruit being the most popular, followed by alcoholic drink, and candy flavors, which is consistent with these flavors’ share of the market nationally [44]. It should be noted that our inclusion criteria included having used a combustible tobacco product in the past week, so the primary pattern of poly-combustible use and the difference in frequency of use of these products could be explained in part by this requirement.

Our findings suggest that YEH are engaging in numerous high-risk tobacco-related behaviors: co-use with marijuana and multiple tobacco products, frequent combustible tobacco use, and use of menthol cigarettes and flavored cigars. Co-using tobacco and cannabis has been linked to a potential increase in exposure to toxic constituents (compared to only using one type of product) [45], more frequent product use, and increased nicotine/marijuana dependence [46–48]. Similarly, poly-tobacco use, frequent combustible product use, and use of flavored products (particularly menthol) are associated with increased nicotine dependence [49–52]. These high-risk behaviors can escalate use, make it difficult to quit, and lead to disproportionate disease burden for people experiencing homelessness.

### Considerations for tobacco cessation intervention

Factors are at play at multiple levels of the social ecology to explain these high-risk behaviors among YEH and to inform interventions targeted for this population. On the individual level, we found that daily combustible tobacco smokers and marijuana users had somewhat unique psychosocial predictors but with similar implications for intervention. Daily combustible tobacco users (47% of the sample) smoked out of habit or boredom and were more likely to have at least one child and to not ignore feelings of pain or discomfort. Daily marijuana users (about one quarter of the sample) were less likely to smoke combustible tobacco due to negative affect, were younger when they initiated tobacco use, and used substances to cope with their housing situation. A national study of young adults found that those who had at least one child were two times as likely to have ever smoked daily than those with no children [53], likely related to added stressors, especially for those without shelter where childcare could also be seen as a competing priority to smoking cessation [21]. Studies also show that young adults who are novelty-seekers and who have more unorganized leisure time are more likely to be daily smokers [53, 54], which could indicate that providing structured activities while at a homeless drop-in center could reduce frequent smoking. For YEH, facilitating access and connection to evidence-based cessation services, such as Quitlines, in the face of daily stressors will be an important initial step toward cessation. Group or individual behavioral cessation counseling for YEH will need to identify stressors and emphasize development of alternative coping strategies.

Also at the individual level, cessation interventions will need to promote recognition/management of triggers to use marijuana and cannabis in relation to one another. We did not assess interest in quitting smoking marijuana, and there is no evidence on marijuana cessation among YEH. However, studies of adults and housing-secure individuals point to the need to address use of both products in tobacco smoking cessation studies. One study of tobacco Quitline callers found that of smokers who also reported currently using marijuana, 43% were interested in quitting marijuana in addition to tobacco [55]. Another study of dual tobacco and marijuana users found compensation of one product when trying to quit the other, with 50% perceiving an increase in their marijuana smoking during tobacco cessation and 62% perceiving an increase in tobacco use during marijuana cessation [56]. Some studies have found reduced tobacco cessation among marijuana users [57, 58]. To develop cessation interventions for YEH, targeting use of both combustible tobacco and marijuana may be necessary. A meta-analysis of interventions targeting co-users found weak evidence for an effect on marijuana cessation and no clear effect on tobacco cessation [59]. Cessation interventions may need to help YEH understand how they use these two products in relation to one another (e.g., to substitute or complement) to elucidate barriers to successful cessation and to better clarify their cessation goals (quitting only tobacco or both products).

At the community/policy level, more frequent combustible tobacco use may be common among YEH because of ease of access due to reduced price and increased availability of cigars in particular (93% of our sample smoked cigars in the past month), which are more likely to be sold in low-income neighborhoods [60, 61]. Menthol cigarettes have been marketed heavily to disadvantaged groups, including young and Black consumers [62], so exposure to such marketing in our sample is likely. Congruently, a national study of brand preferences among young adults found that while Marlboro was the most preferred brand, Newport was more preferred among Black, non-Hispanic and low-income young adults [63]. In the US, federal regulation banning menthol in cigarettes and flavors (including menthol) in cigars has been proposed [64]. Research suggests that policies banning flavors not only have the intended effect of preventing youth initiation, but are also effective in promoting cessation, especially for the 85% of Black smokers who use a menthol brand [65–67]. It remains to be determined what impact a menthol or flavor ban may have on co-use of tobacco and cannabis and use of non-combustible tobacco products. Studies suggest that YEH have misperceptions about the relative risk of non-combustible tobacco compared with cigarettes [16], which could perpetuate the use of combustible products, even if menthol is banned. Correcting for harm misperceptions may be needed through counseling. There is also limited access to evidence-based cessation medication, such as nicotine replacement therapy, for people experiencing homelessness [68]. Given the association of high-risk tobacco use behaviors and increased nicotine dependence [48–50, 52], intervention approaches to directly increase NRT access, access to lower risk nicotine products, or policy changes to restrict nicotine levels in combustible tobacco products should be tested to determine if they may reduce harm and support cessation in this high-risk population [69, 70].

### Innovation, limitations, and future directions

Data are available on tobacco and marijuana use prevalence among other samples of YEH [15], but our study is one of the first to additionally assess flavor use, brands, frequency of use, and predictors of more frequent product use among a sample of YEH. However, there are several limitations to note. First, as our sample was a small convenience sample of YEH in one drop-in center in one city, our results may not generalize to other geographic areas. We also excluded those actively making a current tobacco quit attempt to be consistent with our target population in a future cessation intervention trial, but this may have led to selection bias. Second, measures relied on self-report, so measurement error is possible. Third, another issue possibly affecting measurement is the difficulty in assessing cigars with only tobacco and blunts, which are often conflated [71]. However, providing definitions in the survey questionnaire likely minimized this problem. Future research should further assess co-use of marijuana and tobacco, including frequency/quantity of use, psychosocial contextual factors around co-use, and cessation of these products among YEH.

## Conclusions

Young combustible tobacco users experiencing homelessness engage in high-risk use patterns, including high rates of poly-combustible tobacco use, menthol and other flavored tobacco use, and co-use of tobacco with marijuana. Findings from this study indicate that the highest risk tobacco users are more likely to contend with environmental stressors including having children and also do not ignore or distract themselves from pain, factors that should be considered when targeting cessation support for YEH. Interventions that consider the full context of tobacco and marijuana use are needed to support cessation in this population and to inform policy interventions that promote health equity.

## Supplementary Information

**Additional file 1.**

## Data Availability

The datasets generated and/or analysed during the current study are available in the GitHub repository, https://github.com/nemeth37/TobaccoAndMarijuanaUseYEH.git.

## Abbreviations

CEMS: Children’s Emotion Management Scale
EVP: Electronic vapor product
GED: General educational development
HONC: Hooked on Nicotine Checklist
IQR: Interquartile range
LA: Los Angeles
SOBC: Science of Behavior Change
MAIA: Multidimensional Assessment of Interoceptive Awareness
YEH: Youth experiencing homelessness
US: United States
OR: Odds ratio
CI: Confidence interval
