# Supplementary material for: Characterizing tobacco and marijuana use among youth combustible tobacco users experiencing homelessness – considering product type, brand, flavor, frequency, and higher-risk use patterns and predictors
Source: BMC Public Health. 2022 Apr 25;22:820. doi: 10.1186/s12889-022-13244-3 (PMC9036780; doi:10.1186/s12889-022-13244-3)
Supplement: Supplementary file 1 — Additional file 1. [file 12889_2022_13244_MOESM1_ESM.docx]

**Supplemental Materials**

**Supplemental Table 1. Other Tobacco and Psychosocial Variables**

| **Construct** | **Measures** |
| --- | --- |
| Nicotine dependence | Hooked on Nicotine Checklist: 10-item instrument to identify signals of loss and autonomy among adolescents(34) |
| First use of tobacco | First tobacco product tried: combustible, non-combustible, ENDS, marijuana with tobacco  First regular tobacco product: combustible, non-combustible, ENDS, marijuana with tobacco  Age when first tried tobacco  Age when regularly used tobacco |
| Motivations, temptations, and rewards from smoking | Tobacco Motives Inventory: 15-item instrument to assess social motives, self-enhancement motives, boredom relief motives, and affect regulation motives to smoke(36)  Situational Temptations Inventory for Smoking (Adolescent Form): 10-item instrument to assess temptations from positive social situations, negative affect situations, habit strength, and weight control(35)  Rewards/benefits of smoking: relieves stress, relieves boredom, relieves anxiety, relieves depression and helps lessen sadness, like the taste, helps me socialize and connect with other people, helps me fit in with others around me, can use it as an excuse to get out of a dangerous situation, prevents me from using violence on others, helps me control my anger, it’s calming, it’s comforting, keeps me from feeling hungry, keeps my hands busy, relieves my urge to smoking/helps me feel like I don’t have to smoke, other  Rewards/benefits of quitting smoking: would save money, would improve social relationships, overall health would improve, lung function and lung health would improve, won’t smell like smoke, would decrease risk of cancer and other diseases, food would taste better, won’t have to worry about where my next smoke will come from, other |
| Alcohol use | Lifetime number of days with a drink  Age of first drink  Days drank in the past 30 days  Days binge drank in the past 30 days  Need to smoke when drinking alcohol: never, sometimes, most of the time, every time |
| Interoceptive awareness | SOBC Multidimensional Assessment of Interoceptive Awareness (MAIA): Abbreviated 19-item measure composed of the following 4 subscales: (i) Not-Distracting: the tendency to not ignore or distract oneself from sensations of pain or discomfort; (ii) Attention Regulation: the ability to sustain and control attention to bodily sensation; (iii) Emotional Awareness: the awareness of the connection between bodily sensations and emotional states; and (iv) Self-Regulation: the ability to regulate psychological distress by attention to bodily sensations(38) |
| Coping | SOBC Brief COPE: 28-item instrument assessing coping about one’s housing situation; 14 sub-scales: self-distraction, active coping, denial, substance use, use of emotional support, use of instrumental support, behavioral disengagement, venting, positive reframing, planning, humor, acceptance, religion, self-blame(37) |
| Anger and worry | SOBC Children’s Emotion Management Scale (CEMS) Anger: 11 items related to management of anger(40)  SOBC CEMS Worry: 10 items related to management of worry(39) |

ENDS: electronic nicotine delivery system; SOBC: Science of Behavior Change

**Supplemental Table 2. Associations with Daily Combustible Tobacco Use**

|  | **Combustible Use** | | | |  |
| --- | --- | --- | --- | --- | --- |
|  | **Daily** | | **Non-Daily** | |  |
|  | (n=45) | | (n=51) | | *p-value* |
| **Demographic Characteristics** |  |  |  |  |  |
| Age (*n, %*) |  |  |  |  | 1.000^a^ |
| 14-17 | 1 | 2.22 | 2 | 3.92 |  |
| 18-24 | 44 | 97.78 | 49 | 96.08 |  |
| Age (*mean, SD*) | 21.81 | 2.09 | 21.82 | 1.93 | 0.988^b^ |
| Sex (*n, %*) |  |  |  |  | 0.588^c^ |
| Male | 24 | 53.33 | 30 | 58.82 |  |
| Female | 21 | 46.67 | 21 | 41.18 |  |
| Gender (*n, %*) |  |  |  |  | 0.738^a^ |
| Male | 24 | 53.33 | 28 | 54.90 |  |
| Female | 19 | 42.22 | 20 | 39.22 |  |
| Transgender Female | 0 | 0.00 | 2 | 3.92 |  |
| Transgender Male | 1 | 2.22 | 1 | 1.96 |  |
| Non-binary | 1 | 2.22 | 0 | 0.00 |  |
| Orientation (*n, %*) |  |  |  |  | 0.876^a^ |
| Heterosexual/Straight | 32 | 71.11 | 39 | 76.47 |  |
| Bisexual | 10 | 22.22 | 9 | 17.65 |  |
| Other | 3 | 6.67 | 3 | 5.88 |  |
| Race (*n, %*) |  |  |  |  | 0.939^a^ |
| White | 7 | 15.56 | 8 | 15.69 |  |
| Black | 23 | 51.11 | 28 | 54.90 |  |
| Bi or Multi-racial | 13 | 28.89 | 14 | 27.45 |  |
| Other | 2 | 4.44 | 1 | 1.96 |  |
| Ethnicity (*n, %*) |  |  |  |  | 0.468^a^ |
| Non-Hispanic | 40 | 88.89 | 48 | 94.12 |  |
| Hispanic | 5 | 11.11 | 3 | 5.88 |  |
| Education (*n, %*) |  |  |  |  | 0.355^a^ |
| Less than High School | 18 | 40.00 | 13 | 25.49 |  |
| High School Diploma | 21 | 46.67 | 25 | 49.02 |  |
| GED | 1 | 2.22 | 3 | 5.88 |  |
| More than High School | 5 | 11.11 | 10 | 19.61 |  |
| Children (*n, %*) |  |  |  |  | **0.017^c^** |
| None | 20 | 44.44 | 35 | 68.63 |  |
| 1 or more | 25 | 55.56 | 16 | 31.37 |  |
| Currently Pregnant (*n, %*) |  |  |  |  | 0.054^a^ |
| No | 18 | 85.71 | 18 | 85.71 |  |
| Yes | 0 | 0.00 | 3 | 14.29 |  |
| Don’t Know | 3 | 14.29 | 0 | 0.00 |  |
| Hours Work per Week (*n, %*) |  |  |  |  | 0.103^c^ |
| 0 | 28 | 65.12 | 22 | 43.14 |  |
| 1 – 39 | 7 | 16.28 | 13 | 25.49 |  |
| ≥40 | 8 | 18.60 | 16 | 31.37 |  |
| Where Slept Most Nights (*n, %*) |  |  |  |  | 0.603^c^ |
| With family or friends / Own home | 17 | 37.78 | 14 | 27.45 |  |
| Shelter / Drop-in-center | 10 | 22.22 | 17 | 33.33 |  |
| Group home / Treatment facility / Detention facility | 6 | 13.33 | 7 | 13.73 |  |
| Outside / Car / Tent | 12 | 26.67 | 13 | 25.49 |  |
| **Tobacco Use Characteristics** |  |  |  |  |  |
| HONC (*mean, SD*) | 6.47 | 2.94 | 5.23 | 2.82 | **0.039^b^** |
| Cigar Use (*n, %*) |  |  |  |  | 1.000^a^ |
| Never | 1 | 2.22 | 1 | 1.96 |  |
| Ever | 2 | 4.44 | 3 | 5.88 |  |
| Past 30 Days | 42 | 93.33 | 47 | 92.16 |  |
| Cigarette Use (*n, %*) |  |  |  |  | **0.039^a^** |
| Never | 0 | 0.00 | 5 | 10.00 |  |
| Ever | 1 | 2.22 | 4 | 8.00 |  |
| Past 30 Days | 44 | 97.78 | 41 | 82.00 |  |
| Hookah Use (*n, %*) |  |  |  |  | 0.516^c^ |
| Never | 19 | 42.22 | 22 | 43.14 |  |
| Ever | 15 | 33.33 | 21 | 41.18 |  |
| Past 30 Days | 11 | 24.44 | 8 | 15.69 |  |
| ENDS Use (*n, %*) |  |  |  |  | 0.224^c^ |
| Never | 16 | 35.56 | 11 | 21.57 |  |
| Ever | 13 | 28.89 | 22 | 43.14 |  |
| Past 30 Days | 16 | 35.56 | 18 | 35.29 |  |
| ST Use (*n, %*) |  |  |  |  | 0.340^c^ |
| Never | 32 | 71.11 | 36 | 70.59 |  |
| Ever | 6 | 13.33 | 11 | 21.57 |  |
| Past 30 Days | 7 | 15.56 | 4 | 7.84 |  |
| Marijuana Use (*n, %*) |  |  |  |  | 0.105^c^ |
| Never | 2 | 4.44 | 1 | 1.96 |  |
| Ever | 2 | 4.44 | 9 | 17.65 |  |
| Past 30 Days | 41 | 91.11 | 41 | 80.39 |  |
| Tobacco Use (*n, %*) |  |  |  |  | **0.006^a^** |
| Single Combustible | 0 | 0.00 | 10 | 20.00 |  |
| Poly Combustible | 27 | 60.00 | 21 | 42.00 |  |
| Combustible / ENDS | 11 | 24.44 | 15 | 30.00 |  |
| Combustible / ST | 2 | 4.44 | 1 | 2.00 |  |
| Combustible / ENDS/ ST | 5 | 11.11 | 3 | 6.00 |  |
| Tobacco / Marijuana Use (*n, %*) |  |  |  |  | 0.602^a^ |
| Tobacco Only | 4 | 8.89 | 10 | 19.61 |  |
| Comb. / Marijuana | 24 | 53.33 | 24 | 47.06 |  |
| Comb. / ENDS / Marijuana | 11 | 24.44 | 13 | 25.49 |  |
| Comb. / ST / Marijuana | 2 | 4.44 | 1 | 1.96 |  |
| Comb. / ENDS / ST / Marijuana | 4 | 8.89 | 3 | 5.88 |  |
| First Tobacco Product Tried (*n, %*) |  |  |  |  | 0.620^a^ |
| Combustible | 44 | 97.78 | 46 | 90.20 |  |
| Non-Combustible | 0 | 0.00 | 1 | 1.96 |  |
| ENDS | 0 | 0.00 | 1 | 1.96 |  |
| Marijuana with Tobacco | 1 | 2.22 | 3 | 5.88 |  |
| First Regular Tobacco Product (*n, %*) |  |  |  |  | 0.587^a^ |
| Combustible | 43 | 95.56 | 45 | 88.24 |  |
| Non-Combustible | 0 | 0.00 | 2 | 3.92 |  |
| ENDS | 1 | 2.22 | 1 | 1.96 |  |
| Marijuana with Tobacco | 1 | 2.22 | 3 | 5.88 |  |
| Age when First Tried Tobacco (*mean, SD*) | 13.27 | 3.76 | 14.92 | 3.49 | **0.028^b^** |
| Age when Regularly used Tobacco (*mean, SD*) | 16.42 | 2.43 | 17.08 | 2.46 | 0.193^b^ |
| **Motivations for Smoking** |  |  |  |  |  |
| Social (*median, IQR*) | 7 | 4 – 10 | 8 | 6 – 10 | 0.266^d^ |
| Self Enhancing (*median, IQR*) | 6 | 4 – 9 | 6 | 4 – 10 | 0.814^d^ |
| Boredom Relief (*median, IQR*) | 8 | 6 – 10 | 5 | 3 – 8 | **<0.001^d^** |
| Affect Regulation (*median, IQR*) | 19 | 15 – 21 | 17 | 11 – 21 | 0.164^d^ |
| **Situational Temptations Inventory for Smoking** |  |  |  |  |  |
| Positive Social (*median, IQR*) | -1.35 | -1.73 - -0.96 | -1.35 | -1.73 - -1.15 | 0.246^d^ |
| Negative Affect (*median, IQR*) | -0.86 | -1.21 - -0.52 | -1.03 | -1.38 - -0.86 | **0.024^d^** |
| Habit (*median, IQR*) | -0.81 | -1.00 - -0.42 | -1.00 | -1.38 - -0.81 | **0.001^d^** |
| Weight (*median, IQR*) | -1.12 | -1.12 - -0.54 | -1.12 | -1.12 - -0.73 | 0.936^d^ |
| **Rewards from Quitting and Smoking** |  |  |  |  |  |
| Relieves Stress (*n, %*) | 43 | 95.56 | 41 | 80.39 | **0.025^c^** |
| Relieves Boredom (*n, %*) | 32 | 71.11 | 29 | 56.86 | 0.148^c^ |
| Relieves Anxiety (*n, %*) | 39 | 86.67 | 39 | 76.47 | 0.202^c^ |
| Relieves Depression (*n, %*) | 27 | 60.00 | 27 | 52.94 | 0.487^c^ |
| I like the taste (*n, %*) | 24 | 53.33 | 23 | 45.10 | 0.421^c^ |
| Helps me Socialize (*n, %*) | 19 | 42.22 | 19 | 37.25 | 0.619^c^ |
| Helps me Fit In (*n, %*) | 15 | 33.33 | 15 | 29.41 | 0.679^c^ |
| Excuse to get out of dangerous situations (*n, %*) | 18 | 40.00 | 20 | 40.82 | 0.936^c^ |
| Prevents me from being Violent (*n, %*) | 21 | 46.67 | 23 | 45.10 | 0.878^c^ |
| Helps control my anger (*n, %*) | 29 | 64.44 | 27 | 52.94 | 0.254^c^ |
| It is calming (*n, %*) | 41 | 91.11 | 40 | 78.43 | 0.088^c^ |
| It is comforting (*n, %*) | 36 | 80.00 | 25 | 49.02 | **0.002^c^** |
| Keeps me from feeling hungry (*n, %*) | 15 | 33.33 | 14 | 27.45 | 0.531^c^ |
| Keeps my hands busy (*n, %*) | 29 | 64.44 | 24 | 47.06 | 0.087^c^ |
| Relieves my Urge to Smoke (*n, %*) | 29 | 65.91 | 25 | 49.02 | 0.098^c^ |
| **Alcohol** |  |  |  |  |  |
| Lifetime Days with a Drink (*n, %*) |  |  |  |  | 0.182^c^ |
| 0 | 5 | 11.36 | 6 | 11.76 |  |
| 1 – 9 | 11 | 25.00 | 14 | 27.45 |  |
| 10 – 99 | 12 | 27.27 | 22 | 43.14 |  |
| 100+ | 16 | 36.36 | 9 | 17.65 |  |
| Age of First Drink (*n, %*) |  |  |  |  | 0.494^c^ |
| Never had a drink | 5 | 11.36 | 7 | 13.73 |  |
| 10 or younger | 3 | 6.82 | 8 | 15.69 |  |
| 11 – 14 | 10 | 22.73 | 8 | 15.69 |  |
| 15+ | 26 | 59.09 | 28 | 54.90 |  |
| Days Drank in Past 30 Days (*n, %*) |  |  |  |  | 0.686^c^ |
| 0 Days | 14 | 31.82 | 16 | 32.00 |  |
| 1 or 2 Days | 13 | 29.55 | 16 | 32.00 |  |
| 3 to 5 Days | 7 | 15.91 | 11 | 22.00 |  |
| 6 or More Days | 10 | 22.73 | 7 | 14.00 |  |
| Days Binge Drank in Past 30 Days (*n, %*) |  |  |  |  | **0.043^c^** |
| 0 Days | 10 | 28.57 | 19 | 47.50 |  |
| 1 or 2 Days | 13 | 37.14 | 10 | 25.00 |  |
| 3 to 5 Days | 3 | 8.57 | 8 | 20.00 |  |
| 6 or More Days | 9 | 25.71 | 3 | 7.50 |  |
| Need to Smoke when Drink Alcohol (*n, %*) |  |  |  |  | 0.088^c^ |
| Never | 6 | 15.79 | 12 | 27.27 |  |
| Sometimes | 7 | 18.42 | 8 | 18.18 |  |
| Most of the Time | 5 | 13.16 | 12 | 27.27 |  |
| Every Time | 20 | 52.63 | 12 | 27.27 |  |
| **SOBC MAIA** |  |  |  |  |  |
| Non-Distracting (*median, IQR*) | 1.67 | 1.00 – 3.33 | 1.33 | 0.67 – 2.00 | 0.087^d^ |
| Attention Regulation (*median, IQR*) | 4.14 | 3.14 – 5.00 | 3.71 | 2.86 – 4.71 | 0.310^d^ |
| Emotional Awareness (*median, IQR*) | 4.80 | 4.00 – 5.00 | 4.80 | 4.20 – 5.00 | 0.658^d^ |
| Self-Regulation (*median, IQR*) | 4.00 | 2.50 – 5.00 | 4.00 | 3.25 – 4.75 | 0.569^d^ |
| **SOBC Brief COPE** |  |  |  |  |  |
| Self-distracting (*median, IQR*) | 5 | 4 – 8 | 5 | 4 – 7 | 0.493^d^ |
| Active Coping (*median, IQR*) | 7 | 5 – 8 | 7 | 6 – 8 | 0.895^d^ |
| Denial (*median, IQR*) | 4 | 2 – 7 | 4 | 3 – 7 | 0.383^d^ |
| Substance (*median, IQR*) | 5 | 2 – 7 | 4 | 3 – 6 | 0.327^d^ |
| Emotional (*median, IQR*) | 4 | 4 – 6 | 6 | 4 – 7 | 0.130^d^ |
| Instrumental (*median, IQR*) | 5 | 4 – 6 | 6 | 4 – 7 | 0.252^d^ |
| Disengagement (*median, IQR*) | 3 | 2 – 5 | 4 | 2 – 5 | 0.502^d^ |
| Venting (*median, IQR*) | 4 | 3 – 6 | 4 | 3 – 6 | 0.196^d^ |
| Positive Frame (*median, IQR*) | 6 | 4 – 8 | 6 | 5 – 8 | 0.436^d^ |
| Planning (*median, IQR*) | 6 | 5 – 8 | 7 | 5 – 8 | 0.529^d^ |
| Humor (*median, IQR*) | 5 | 2 – 7.5 | 5 | 2 – 7 | 0.756^d^ |
| Acceptance (*median, IQR*) | 6.5 | 5 – 8 | 6 | 4 – 8 | 0.253^d^ |
| Religion (*median, IQR*) | 4 | 2 – 6 | 6 | 4 – 6 | 0.177^d^ |
| Self Blame (*median, IQR*) | 5 | 3 – 8 | 5.5 | 4 – 8 | 0.158^d^ |
| **SOBC CEMS: Anger** |  |  |  |  |  |
| Inhibition (*median, IQR*) | 9 | 8 – 10 | 9 | 8 – 11 | 0.327^d^ |
| Dysregulation (*median, IQR*) | 5 | 3 – 7 | 5 | 4 – 6 | 0.619^d^ |
| Coping (*median, IQR*) | 9 | 8 – 10.5 | 9 | 8 – 11 | 0.682^d^ |
| Overall (*median, IQR*) | 23 | 21 – 25 | 23 | 21 – 26 | 0.747^d^ |
| **SOBC CEMS: Worry** |  |  |  |  |  |
| Inhibition (*median, IQR*) | 9 | 8 – 11 | 9 | 8 – 11 | 0.229^d^ |
| Dysregulation (*median, IQR*) | 5 | 4 – 6 | 5 | 4 – 7 | 0.376^d^ |
| Coping (*median, IQR*) | 7 | 5 – 7 | 6 | 6 – 7 | 0.946^d^ |
| Overall (*median, IQR*) | 20 | 18 – 23 | 21 | 19 – 23 | 0.267^d^ |

^a^ Fisher exact test

^b^ t-test

^c^ Chi-square test

^d^ Wilcoxon rank-sum test

**Supplemental Table 3. Associations with Daily Marijuana Use (among past 30-day marijuana users)**

|  | **Past 30-day Marijuana Use** | | | |  |
| --- | --- | --- | --- | --- | --- |
|  | **Daily** | | **Not Daily** | |  |
|  | (n=26) | | (n=56) | | *p-value* |
| **Demographic Characteristics** |  |  |  |  |  |
| Age (*n, %*) |  |  |  |  | 0.235^a^ |
| 14-17 | 2 | 7.69 | 1 | 1.79 |  |
| 18-24 | 24 | 92.31 | 55 | 98.21 |  |
| Age (*mean, SD*) | 21.71 | 2.19 | 21.76 | 1.98 | 0.927^b^ |
| Sex (*n, %*) |  |  |  |  | 0.180^c^ |
| Male | 18 | 69.23 | 30 | 53.57 |  |
| Female | 8 | 30.77 | 26 | 46.43 |  |
| Gender (*n, %*) |  |  |  |  | 0.582^a^ |
| Male | 18 | 69.23 | 28 | 50.00 |  |
| Female | 8 | 30.77 | 23 | 41.07 |  |
| Transgender Female | 0 | 0.00 | 2 | 3.57 |  |
| Transgender Male | 0 | 0.00 | 2 | 3.57 |  |
| Non-binary | 0 | 0.00 | 1 | 1.79 |  |
| Gender (*n, %)* |  |  |  |  | 0.263^a^ |
| Male | 18 | 69.23 | 29 | 51.79 |  |
| Female | 8 | 30.77 | 24 | 42.86 |  |
| Non-binary | 0 | 0.00 | 3 | 5.36 |  |
| Orientation (*n, %*) |  |  |  |  | 0.783^a^ |
| Heterosexual/Straight | 20 | 76.92 | 40 | 71.43 |  |
| Bisexual | 5 | 19.23 | 11 | 19.64 |  |
| Other | 1 | 3.85 | 5 | 8.93 |  |
| Race (*n, %*) |  |  |  |  | 0.207^a^ |
| White | 3 | 11.54 | 10 | 17.86 |  |
| Black | 11 | 42.31 | 32 | 57.14 |  |
| Bi or Multi-racial | 10 | 38.46 | 13 | 23.21 |  |
| Other | 2 | 7.69 | 1 | 1.79 |  |
| Ethnicity (*n, %*) |  |  |  |  | 1.000^a^ |
| Non-Hispanic | 24 | 92.31 | 50 | 89.29 |  |
| Hispanic | 2 | 7.69 | 6 | 10.71 |  |
| Education (*n, %*) |  |  |  |  | 0.611^a^ |
| Less than High School | 10 | 38.46 | 17 | 30.36 |  |
| High School Diploma | 13 | 50.00 | 27 | 48.21 |  |
| GED | 0 | 0.00 | 4 | 7.14 |  |
| More than High School | 3 | 11.54 | 8 | 14.29 |  |
| Children (*n, %*) |  |  |  |  | 0.707^c^ |
| None | 16 | 61.54 | 32 | 57.14 |  |
| 1 or more | 10 | 38.46 | 24 | 42.86 |  |
| Currently Pregnant (*n, %*) |  |  |  |  | 0.678^a^ |
| No | 7 | 87.50 | 23 | 88.46 |  |
| Yes | 0 | 0.00 | 2 | 7.69 |  |
| Don’t Know | 1 | 12.50 | 1 | 3.85 |  |
| Hours Work per Week (*n, %*) |  |  |  |  | 0.149^c^ |
| 0 | 11 | 44.00 | 31 | 56.36 |  |
| 1 – 39 | 9 | 36.00 | 9 | 16.36 |  |
| ≥40 | 5 | 20.00 | 15 | 27.27 |  |
| Where Slept Most Nights (*n, %*) |  |  |  |  | 0.777^c^ |
| With family or friends / Own home | 8 | 30.77 | 20 | 35.71 |  |
| Shelter / Drop-in-center | 6 | 23.08 | 16 | 28.57 |  |
| Group home / Treatment facility / Detention facility | 4 | 15.38 | 5 | 8.93 |  |
| Outside / Car / Tent | 8 | 30.77 | 15 | 26.79 |  |
| **Tobacco Use Characteristics** |  |  |  |  |  |
| HONC (*mean, SD*) | 4.73 | 3.18 | 6.35 | 2.66 | **0.017^b^** |
| Cigar Use (*n, %*) |  |  |  |  | 0.100^a^ |
| Never | 1 | 3.85 | 1 | 1.79 |  |
| Ever | 3 | 11.54 | 1 | 1.79 |  |
| Past 30 Days | 22 | 84.62 | 54 | 96.43 |  |
| Cigarette Use (*n, %*) |  |  |  |  | 1.000^a^ |
| Never | 0 | 0.00 | 1 | 1.82 |  |
| Ever | 0 | 0.00 | 2 | 3.64 |  |
| Past 30 Days | 26 | 100.00 | 52 | 94.55 |  |
| Hookah Use (*n, %*) |  |  |  |  | 0.967^c^ |
| Never | 10 | 38.46 | 23 | 41.07 |  |
| Ever | 10 | 38.46 | 20 | 35.71 |  |
| Past 30 Days | 6 | 23.08 | 13 | 23.21 |  |
| ENDS Use (*n, %*) |  |  |  |  | 0.396^c^ |
| Never | 8 | 30.77 | 16 | 28.57 |  |
| Ever | 6 | 23.08 | 21 | 37.50 |  |
| Past 30 Days | 12 | 46.15 | 19 | 33.93 |  |
| ST Use (*n, %*) |  |  |  |  | 0.721^c^ |
| Never | 18 | 69.23 | 38 | 67.86 |  |
| Ever | 4 | 15.38 | 12 | 21.43 |  |
| Past 30 Days | 4 | 15.38 | 6 | 10.71 |  |
| Tobacco Use (*n, %*) |  |  |  |  | 0.773^a^ |
| Single Combustible | 0 | 0.00 | 3 | 5.45 |  |
| Poly Combustible | 13 | 50.00 | 31 | 56.36 |  |
| Combustible / ENDS | 9 | 34.62 | 15 | 27.27 |  |
| Combustible / ST | 1 | 3.85 | 2 | 3.64 |  |
| Combustible / ENDS/ ST | 3 | 11.54 | 4 | 7.27 |  |
| Tobacco / Marijuana Use (*n, %*) |  |  |  |  | 0.680^c^ |
| Comb. / Marijuana | 13 | 50.00 | 35 | 62.50 |  |
| Comb. / ENDS / Marijuana | 9 | 34.62 | 15 | 26.79 |  |
| Comb. / ST / Marijuana | 1 | 3.85 | 2 | 3.57 |  |
| Comb. / ENDS / ST / Marijuana | 3 | 11.54 | 4 | 7.14 |  |
| First Tobacco Product Tried (*n, %*) |  |  |  |  | **0.048^a^** |
| Combustible | 22 | 84.62 | 55 | 98.21 |  |
| Non-Combustible | 1 | 3.85 | 0 | 0.00 |  |
| ENDS | 1 | 3.85 | 0 | 0.00 |  |
| Marijuana with Tobacco | 2 | 7.69 | 1 | 1.79 |  |
| First Regular Tobacco Product (*n, %*) |  |  |  |  | 0.182^a^ |
| Combustible | 23 | 88.46 | 53 | 94.64 |  |
| Non-Combustible | 1 | 3.85 | 0 | 0.00 |  |
| ENDS | 0 | 0.00 | 2 | 3.57 |  |
| Marijuana with Tobacco | 2 | 7.69 | 1 | 1.79 |  |
| Age when First Tried Tobacco (*mean, SD*) | 12.62 | 3.52 | 14.18 | 3.52 | 0.065^b^ |
| Age when Regularly used Tobacco (*mean, SD*) | 16.23 | 2.18 | 16.77 | 2.23 | 0.310^b^ |
| **Motivations for Smoking** |  |  |  |  |  |
| Social (*median, IQR*) | 7.50 | 5.00 – 10.00 | 8.00 | 4.00 – 10.00 | 0.912^d^ |
| Self Enhancing (*median, IQR*) | 6.00 | 4.00 – 8.00 | 6.00 | 5.00 – 10.00 | 0.755^d^ |
| Boredom Relief (*median, IQR*) | 7.50 | 4.00 – 10.00 | 7.00 | 4.00 – 8.00 | 0.454^d^ |
| Affect Regulation (*median, IQR*) | 16.00 | 11.00 – 21.00 | 19.00 | 15.00 – 21.00 | 0.263^d^ |
| **Situational Temptations Inventory for Smoking** |  |  |  |  |  |
| Positive Social (*median, IQR*) | -1.35 | -1.92 - -1.15 | -1.35 | -1.35 - -0.96 | **0.045^d^** |
| Negative Affect (*median, IQR*) | -1.21 | -1.55 - -0.69 | -0.86 | -1.21 - -0.69 | **0.015^d^** |
| Habit (*median, IQR*) | -1.00 | -1.38 - -0.81 | -1.00 | -1.19 - -0.62 | 0.164^d^ |
| Weight (*median, IQR*) | -1.12 | -1.12 - -0.92 | -0.92 | -1.12 - -0.73 | 0.215^d^ |
| **Rewards from Quitting and Smoking** |  |  |  |  |  |
| Relieves Stress (*n, %*) | 21 | 80.77 | 52 | 92.86 | 0.134^a^ |
| Relieves Boredom (*n, %*) | 18 | 69.23 | 35 | 62.50 | 0.553^c^ |
| Relieves Anxiety (*n, %*) | 19 | 73.08 | 48 | 85.71 | 0.221^a^ |
| Relieves Depression (*n, %*) | 13 | 50.00 | 32 | 57.14 | 0.545^c^ |
| I like the taste (*n, %*) | 11 | 42.31 | 29 | 51.79 | 0.424^c^ |
| Helps me Socialize (*n, %*) | 8 | 30.77 | 24 | 42.86 | 0.296^c^ |
| Helps me Fit In (*n, %*) | 6 | 23.08 | 20 | 35.71 | 0.253^c^ |
| Excuse to get out of dangerous situations (*n, %*) | 12 | 46.15 | 20 | 37.04 | 0.456^c^ |
| Prevents me from being Violent (*n, %*) | 12 | 46.15 | 29 | 51.79 | 0.635^c^ |
| Helps control my anger (*n, %*) | 15 | 57.69 | 35 | 62.50 | 0.678^c^ |
| It is calming (*n, %*) | 19 | 73.08 | 51 | 91.07 | **0.045^a^** |
| It is comforting (*n, %*) | 14 | 53.85 | 41 | 73.21 | 0.082^c^ |
| Keeps me from feeling hungry (*n, %*) | 8 | 30.77 | 16 | 28.57 | 0.839^c^ |
| Keeps my hands busy (*n, %*) | 12 | 46.15 | 36 | 64.29 | 0.121^c^ |
| Relieves my Urge to Smoke (*n, %*) | 11 | 42.31 | 33 | 60.00 | 0.136^c^ |
| **Alcohol** |  |  |  |  |  |
| Lifetime Days with a Drink (*n, %*) |  |  |  |  | 0.133^c^ |
| 0 | 3 | 11.54 | 6 | 10.91 |  |
| 1 – 9 | 9 | 34.62 | 11 | 20.00 |  |
| 10 – 99 | 5 | 19.23 | 25 | 45.45 |  |
| 100+ | 9 | 34.62 | 13 | 23.64 |  |
| Age of First Drink (*n, %*) |  |  |  |  | 0.275^a^ |
| Never had a drink | 3 | 11.54 | 6 | 10.91 |  |
| 10 or younger | 4 | 15.38 | 7 | 12.73 |  |
| 11 – 14 | 8 | 30.77 | 8 | 14.55 |  |
| 15+ | 11 | 42.31 | 34 | 61.82 |  |
| Days Drank in Past 30 Days (*n, %*) |  |  |  |  | 0.638^a^ |
| 0 Days | 7 | 26.92 | 17 | 30.91 |  |
| 1 or 2 Days | 8 | 30.77 | 19 | 34.55 |  |
| 3 to 5 Days | 4 | 15.38 | 11 | 20.00 |  |
| 6 or More Days | 7 | 26.92 | 8 | 14.55 |  |
| Days Binge Drank in Past 30 Days (*n, %*) |  |  |  |  | 0.416^a^ |
| 0 Days | 7 | 31.82 | 19 | 43.18 |  |
| 1 or 2 Days | 7 | 31.82 | 14 | 31.82 |  |
| 3 to 5 Days | 2 | 9.09 | 6 | 13.64 |  |
| 6 or More Days | 6 | 27.27 | 5 | 11.36 |  |
| Need to Smoke when Drink Alcohol (*n, %*) |  |  |  |  | 0.142^a^ |
| Never | 6 | 26.09 | 7 | 14.58 |  |
| Sometimes | 2 | 8.70 | 12 | 25.00 |  |
| Most of the Time | 7 | 30.43 | 7 | 14.58 |  |
| Every Time | 8 | 34.78 | 22 | 45.83 |  |
| **SOBC MAIA** |  |  |  |  |  |
| Non-Distracting (*median, IQR*) | 1.67 | 0.67 – 3.33 | 1.33 | 0.83 – 2.00 | 0.108^d^ |
| Attention Regulation (*median, IQR*) | 4.29 | 3.14 – 5.00 | 3.71 | 2.57 – 4.57 | 0.129^d^ |
| Emotional Awareness (*median, IQR*) | 4.80 | 4.00 – 5.00 | 4.60 | 4.00 – 5.00 | 0.438^d^ |
| Self-Regulation (*median, IQR*) | 4.00 | 3.00 – 5.00 | 4.00 | 2.75 – 4.75 | 0.394^d^ |
| **SOBC Brief COPE** |  |  |  |  |  |
| Self-distracting (*median, IQR*) | 5.00 | 4.00 – 7.00 | 5.00 | 4.00 – 7.00 | 0.709^d^ |
| Active Coping (*median, IQR*) | 8.00 | 6.00 – 8.00 | 7.00 | 5.00 – 8.00 | 0.052^d^ |
| Denial (*median, IQR*) | 4.00 | 2.00 – 6.00 | 5.00 | 3.00 – 7.00 | 0.224^d^ |
| Substance (*median, IQR*) | 6.00 | 4.00 – 8.00 | 5.00 | 3.00 – 6.0 | 0.159^d^ |
| Emotional (*median, IQR*) | 6.00 | 4.00 – 8.00 | 5.50 | 4.00 – 7.00 | 0.803^d^ |
| Instrumental (*median, IQR*) | 5.00 | 4.00 – 8.00 | 5.00 | 4.00 – 7.00 | 0.489^d^ |
| Disengagement (*median, IQR*) | 3.00 | 2.00 – 4.00 | 4.00 | 2.00 – 5.00 | 0.367^d^ |
| Venting (*median, IQR*) | 4.00 | 3.00 – 7.00 | 5.00 | 3.00 - 6.00 | 0.605^d^ |
| Positive Frame (*median, IQR*) | 6.00 | 5.00 – 7.00 | 6.00 | 4.50 – 7.50 | 0.972^d^ |
| Planning (*median, IQR*) | 8.00 | 6.00 – 8.00 | 6.00 | 5.00 – 8.00 | 0.134^d^ |
| Humor (*median, IQR*) | 5.00 | 3.00 – 8.00 | 5.00 | 2.00 – 7.00 | 0.548^d^ |
| Acceptance (*median, IQR*) | 7.00 | 6.00 – 8.00 | 6.00 | 4.00 – 8.00 | 0.076^d^ |
| Religion (*median, IQR*) | 5.50 | 3.00 – 8.00 | 5.00 | 2.00 – 6.00 | 0.396^d^ |
| Self Blame (*median, IQR*) | 5.00 | 4.00 – 7.00 | 5.00 | 4.00 – 8.00 | 0.870^d^ |
| **SOBC CEMS: Anger** |  |  |  |  |  |
| Inhibition (*median, IQR*) | 9.00 | 7.00 – 11.00 | 9.00 | 8.00 – 10.00 | 0.686^d^ |
| Dysregulation (*median, IQR*) | 5.00 | 3.00 – 6.00 | 5.00 | 4.00 – 7.00 | 0.478^d^ |
| Coping (*median, IQR*) | 9.00 | 8.00 – 10.00 | 8.00 | 7.00 – 10.00 | 0.468^d^ |
| Overall (*median, IQR*) | 23.00 | 21.00 – 25.00 | 22.00 | 20.00 – 26.00 | 0.692^d^ |
| **SOBC CEMS: Worry** |  |  |  |  |  |
| Inhibition (*median, IQR*) | 9.00 | 7.00 – 11.00 | 9.00 | 8.00 – 11.00 | 0.602^d^ |
| Dysregulation (*median, IQR*) | 4.00 | 3.00 – 6.00 | 5.00 | 4.00 – 6.00 | **0.045^d^** |
| Coping (*median, IQR*) | 6.00 | 5.00 – 7.00 | 6.50 | 6.00 – 7.00 | 0.493^d^ |
| Overall (*median, IQR*) | 19.00 | 17.00 – 23.00 | 21.00 | 19.00 – 23.00 | 0.134^d^ |

^a^ Fisher exact test

^b^ t-test

^c^ Chi-square test

^d^ Wilcoxon rank-sum test

**Supplemental Table 4. Usual Flavor^a^ Among Ever Users Who Reported a Usual Brand**

|  | **Cigarettes (n=81)** | | **Cigars (n=76)** | | **EVP (n=18)** | | **Smokeless (n=12)** | |
| --- | --- | --- | --- | --- | --- | --- | --- | --- |
|  | **n** | **%** | **n** | **%** | **n** | **%** | **n** | **%** |
| No Usual Flavor | 20 | 24.69 | 39 | 51.32 | 1 | 5.56 | 0 | 0 |
| Menthol or mint | 59 | 72.84 | 3 | 3.95 | 2 | 11.11 | 9 | 75.00 |
| Fruit | 2 | 2.47 | 12 | 15.79 | 10 | 55.56 | 3 | 25.00 |
| An alcoholic drink (such as wine, cognac, margarita pina colada, peach schnapps, or other cocktails) | 0 | 0 | 10 | 13.16 | 0 | 0 | 0 | 0 |
| Candy or other sweets | 0 | 0 | 8 | 10.53 | 3 | 16.67 | 0 | 0 |
| Coffee | 0 | 0 | 1 | 1.32 | 1 | 5.56 | 0 | 0 |
| Vanilla | 0 | 0 | 1 | 1.32 | 0 | 0 | 0 | 0 |
| Multiple flavors | 0 | 0 | 1 | 1.32 | 0 | 0 | 0 | 0 |
| Don’t Know | 0 | 0 | 0 | 0 | 1 | 5.56 | 0 | 0 |
| Missing | 0 | 0 | 1 | 1.32 | 0 | 0 | 0 | 0 |

^a^ other flavors that we assessed but were not used by our sample were clove/spice, chocolate, tobacco, cola, and other

EVP: electronic vapor product

**Supplemental Figure 1. Usual Brand Reported in the Past 30 Days**

EVP: electronic vapor product
